# Infections by human gastrointestinal helminths are associated with changes in faecal microbiota diversity and composition

**DOI:** 10.1371/journal.pone.0184719

**Published:** 2017-09-11

**Authors:** Timothy P. Jenkins, Yasara Rathnayaka, Piyumali K. Perera, Laura E. Peachey, Matthew J. Nolan, Lutz Krause, Rupika S. Rajakaruna, Cinzia Cantacessi

**Affiliations:** 1 Department of Veterinary Medicine, University of Cambridge, Cambridge, United Kingdom; 2 Department of Zoology, University of Peradeniya, Peradeniya, Sri Lanka; 3 Royal Veterinary College, University of London, North Mymms, United Kingdom; 4 The University of Queensland Diamantina Institute, Translational Research Institute, Woolloongabba, Australia; Universidade de Aveiro, PORTUGAL

## Abstract

Investigations of the impact that patent infections by soil-transmitted gastrointestinal nematode parasites exert on the composition of the host gut commensal flora are attracting growing interest by the scientific community. However, information collected to date varies across experiments, and further studies are needed to identify consistent relationships between parasites and commensal microbial species. Here, we explore the qualitative and quantitative differences between the microbial community profiles of cohorts of human volunteers from Sri Lanka with patent infection by one or more parasitic nematode species (*H+*), as well as that of uninfected subjects (*H-*) and of volunteers who had been subjected to regular prophylactic anthelmintic treatment (*Ht*). High-throughput sequencing of the bacterial 16S rRNA gene, followed by bioinformatics and biostatistical analyses of sequence data revealed no significant differences in alpha diversity (Shannon) and richness between groups (P = 0.65, P = 0.13 respectively); however, beta diversity was significantly increased in *H+* and *Ht* when individually compared to *H-*volunteers (P = 0.04). Among others, bacteria of the families *Verrucomicrobiaceae* and *Enterobacteriaceae* showed a trend towards increased abundance in *H+*, whereas the *Leuconostocaceae* and *Bacteroidaceae* showed a relative increase in *H*- and *Ht* respectively. Our findings add valuable knowledge to the vast, and yet little explored, research field of parasite—microbiota interactions and will provide a basis for the elucidation of the role such interactions play in pathogenic and immune-modulatory properties of parasitic nematodes in both human and animal hosts.

## Introduction

More than 1 billion people worldwide are estimated to be infected by gastrointestinal (GI) soil-transmitted helminths, including the roundworm *Ascaris lumbricoides*, the whipworm *Trichuris trichiura* and the hookworms *Necator americanus* and *Ancylostoma duodenale* [1]. Infections by these nematodes alone are estimated to cause the loss of 4.98 million disability-adjusted life years (DALYs) globally [2], mainly affecting areas of developing countries characterised by suboptimal standards of sanitation and hygiene [3]. Despite global efforts to control infections by these parasites *via* mass drug administration (MDA), repeated exposure to infective larvae and high re-infection rates in at-risk populations make interruption of the life cycles of these nematodes and their elimination difficult to achieve [4, 5]. These challenges, together with the realistic threat of emerging drug resistance [6] drive the continual search for new, integrated strategies to control these diseases, based on a thorough understanding of the fundamental biology and epidemiology of these pathogens and their interactions with the human host [7]. Recently, studies of the intimate mechanisms that regulate the relationships between GI soil-transmitted nematodes and their vertebrate hosts have involved investigations of the impact that patent parasite infections exert on the composition of the gut commensal flora and relative abundance of individual bacterial groups [8–10]. The increased attention towards detailed explorations of parasite-microbiota interactions stems from knowledge that the gut commensal flora plays several key essential roles in human health, including nutrient metabolism, protection against pathogens and regulation of both innate and adaptive immune responses [11, 12]. Therefore, given that GI nematodes and the gut microbial flora share the same environment within the human host, it is plausible that parasite-microbiota interactions impact substantially on the health and homeostasis of helminth-infected individuals [10]. For instance, GI nematodes and the microbiota compete for host nutrients while, in parallel, the known immune-modulatory properties of a range of parasites may translate into dramatic modifications of the mucosal and systemic immunity to the resident bacteria [10]. The effects that GI nematode infections exert on the gut commensal flora of vertebrate hosts have long been neglected; however, recent studies have contributed preliminary information to this little-known field of research, mainly driven by the need to better understand the factors that determine the immune-modulatory properties of selected species of parasitic nematodes [13–17]. Our group has recently attempted to determine the impact that experimental infections by the human hookworm, *N*. *americanus*, exert on the composition of the gut microbiota of human volunteers [15, 17, 18]. While no shifts in the relative abundance of individual bacterial taxa were observed over the course of these studies, increases in microbial species richness and diversity were detected following experimental infections [15, 17, 18]. However, these studies, conducted under controlled experimental settings and with a known number of infective larvae, are unlikely to represent ‘real-world’ infections (caused by the simultaneous presence of multiple parasite species with varying infection burdens). Thus far, to the best of our knowledge, only two studies have evaluated differences in the composition of the gut microbiota of human subjects naturally infected by GI nematodes [8, 9], with contrasting results. Indeed, while in the first study, Cooper and colleagues [8] detected a reduction in faecal bacterial diversity in Ecuadorean school children naturally infected by *T*. *trichiura* and *A*. *lumbricoides* compared to uninfected children or children solely infected by the former, a study by Lee et al. [9], reported a greater richness in the faecal microbiota of a cohort of indigenous Malaysian volunteers infected by multiple GI nematodes (i.e. hookworms, whipworms and roundworms) when compared with that of a group of uninfected subjects from New York. This data highlights the need for additional explorations of the impact that natural patent infections exert on the gut microbiota of infected human subjects. In addition, in the studies by Cooper et al. [8] and Lee et al. [9], the microbial profiling of helminth-infected and uninfected subjects with that of volunteers subjected to regular anthelmintic treatment was not examined. Given the widespread use of MDA in parasite-endemic areas [5], the possible consequences of regular use of chemotherapeutics on the composition of the host gut commensal flora deserves further investigation. Amongst the developing countries in which MDA is in use, Sri Lanka provides a suitable setting for such a study, given a 29% prevalence estimate for soil-transmitted helminths (including GI nematodes) in school children [19] and the implementation of mass deworming programmes since 1994 [20]. Therefore, in this study, we explore the qualitative and quantitative differences between the microbial community profiles of human volunteers (from diverse Sri Lankan communities) infected by one or more GI nematode species, and compare the gut microbial profiles of these subjects with those from a cohort of volunteers from the same geographical area who had been subjected to regular prophylactic anthelmintic treatment.

## Materials and methods

### Ethics statement

This study was approved and carried out in strict accordance and compliance with the guidelines of the Institutional Ethical Review Committee, Faculty of Medicine, University of Peradeniya, Sri Lanka (Research Project No. 2015/EC/58). Written informed consent was obtained from all subjects enrolled in the study.

### Study area and characteristics of the population

**Fig 1:**
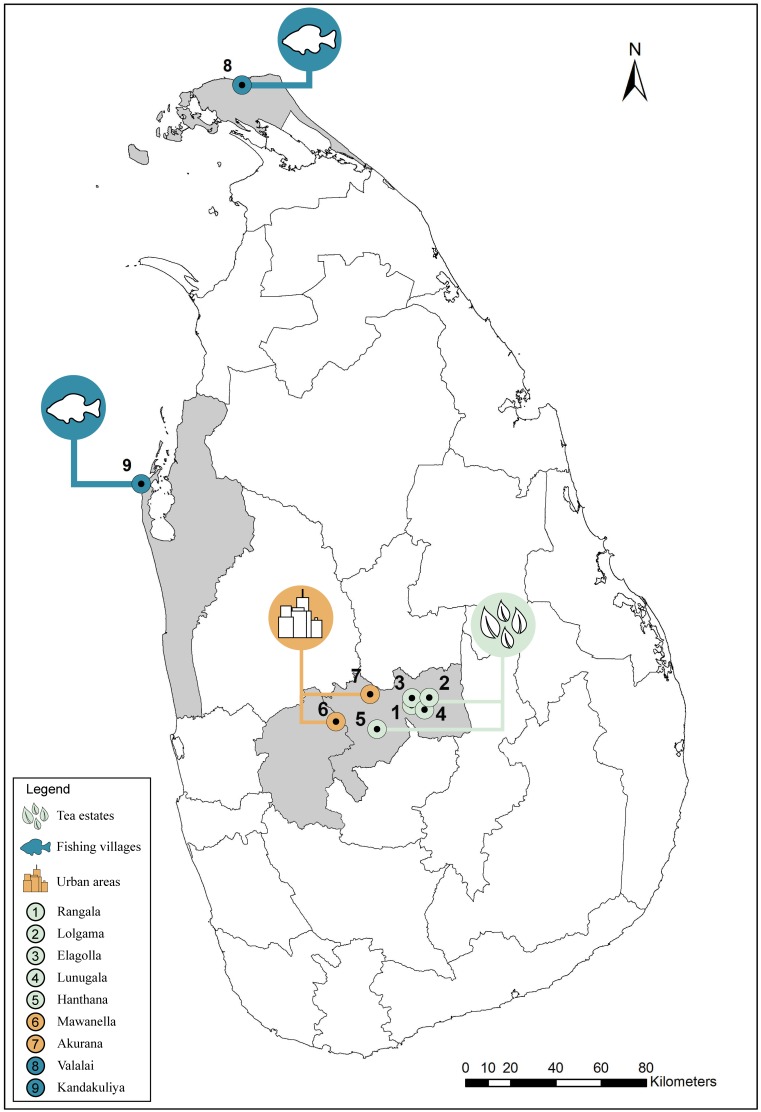
Sampling locations in Sri Lanka. Numbers 1–9 represent the villages Rangala, Lolgama, Elagolla, Lunugala, Hanthana, Mawanella, Akurana (in Kandy District), Valalai (in Jaffna District) and Kandakuliya (in Puttalam District), respectively.

A total number of 76 subjects from nine villages in four districts of Sri Lanka were screened for the presence of patent infections by GI nematodes (Fig 1). Participants were distributed as follows:

- 45 subjects from the Kandy district, villages of Rangala, Lolgama, Elagolla, Lunugala, Hanthana, Akurana;
- 10 from the Jaffna district, Valalai village;
- 15 from the Puttalam district, Kandakuliya village;
- 6 from the Kegalle district, Mawanella village (Fig 1).

Subjects were both men and women, of varying ages and social background, with no reported symptoms of GI disease or any other concomitant diseases, and who had not received antibiotic treatment over at least 6 months prior to the study (Table 1). Participants from the villages of Rangala, Lolgama, Elagolla, Lunugala and Hanthana were workers in tea estates, while those from Kandakuliya and Valalai were fishermen (cf. Fig 1). Subjects from Mawanella and Akurana were living in congested urban areas characterised by poor sanitary living conditions. All participants were interviewed directly by YR, PKP and RSR using a standardised, pre-tested questionnaire aimed to identify means of access to water, knowledge of sanitary and hygiene standards, availability of and access to health care facilities, awareness of risks of infection by GI helminths, and frequency of anthelmintic treatments. A copy of the questionnaire is provided in S1 Fig.

**Table 1:** Village, age (years), gender of helminth-positive (*H*+), helminth-negative (*H*-) and helminth-negative but regularly treated (*Ht*) subjects enrolled in this study. The parasite species infecting *H+* volunteers and compound administered to *Ht* volunteers are also indicated (NA = not applicable; NP = not provided).

ID	Village	Age (years)	Gender	Parasite species / Drug
***H+***				
*H+* 01	Hanthana	1–18	Male	*Ascaris*
*H+* 02	Hanthana	1–18	Male	*Ascaris*
*H+* 03	Hanthana	1–18	Female	*Ascaris*
*H+* 04	Hanthana	1–18	Female	*Ascaris*
*H+* 05	Hanthana	1–18	Female	*Ascaris*
*H+* 06	Hanthana	1–18	Male	*Ascaris*
*H+* 07	Kandakuliya	1–18	Female	*Trichuris*
*H+* 08	Mawanella	1–18	Male	*Hookworm*
*H+* 09	Mawanella	51+	Female	*Hookworm*
*H+* 10	Rangala	19–50	Male	*Hookworm*
*H+* 11	Rangala	1–18	Female	*Hookworm*
***H-***				
*H-* 01	Hanthana	1–18	Male	NA
*H-* 02	Hanthana	1–18	Male	NA
*H-* 03	Hanthana	1–18	Male	NA
*H-* 04	Hanthana	1–18	Female	NA
*H-* 05	Hanthana	1–18	Male	NA
*H-* 06	Hanthana	1–18	Male	NA
*H-* 07	Hanthana	1–18	Male	NA
*H-* 08	Hanthana	1–18	Female	NA
*H-* 09	Hanthana	NP	NP	NA
*H-* 10	Hanthana	1–18	Male	NA
*H-* 11	Hanthana	1–18	Male	NA
***Ht***				
*Ht* 01	Akurana	1–18	Female	Pyrantel pamoate
*Ht* 02	Akurana	51+	Female	Pyrantel pamoate
*Ht* 03	Kandakuliya	1–18	Female	Pyrantel pamoate
*Ht* 04	Kandakuliya	19–50	Male	Pyrantel pamoate
*Ht* 05	Kandakuliya	1–18	Female	Pyrantel pamoate
*Ht* 06	Kandakuliya	1–18	Female	Pyrantel pamoate
*Ht* 07	Mawanella	19–50	Female	Pyrantel pamoate
*Ht* 08	Mawanella	1–18	Female	Pyrantel pamoate
*Ht* 09	Mawanella	1–18	Male	Pyrantel pamoate
*Ht* 10	Valalai	19–50	Female	Pyrantel pamoate
*Ht* 11	Valalai	1–18	Male	Pyrantel pamoate

### Sample collection and parasitological analyses

Each volunteer was asked to provide a fresh stool sample for parasitological analyses. The presence of nematode eggs/larvae in each sample was assessed using a modified sucrose flotation method described previously [21]. Stool samples were refrigerated and transported to the laboratory for processing. For each sample, approximately 3 g of faeces were measured, mixed with distilled water in a capped centrifuge tube to a final volume of 15 ml. The mixtures were stirred thoroughly using a wooden applicator and centrifuged at 2045 g for 20 mins at room temperature (~27°C). Following centrifugation, the supernatants were discarded and the resulting pellets were re-suspended in distilled water and centrifuged (twice) until clear supernatants were obtained. The pellets were then emulsified using saturated sucrose solution, mixed thoroughly, and centrifuged for 20 min at 2045 g. Approximately 5 ml of the top meniscus of the resulting suspensions were collected in a centrifuge tube, mixed with distilled water up to a final volume of 15 ml and centrifuged for 10 min at 1370 g. This procedure was repeated and 1 ml of each suspension with the pellet was transferred to 1.5 ml eppendorf^®^ tubes using a Pasteur pipette. Distilled water was added to a final volume of 1.5 ml and the tubes were centrifuged at 1150 g for 10 min. The clear supernatants were decanted and microscope slides were prepared using the remaining 0.5 ml pellets and examined under a light microscope. Helminth eggs were identified using established morphological keys [22].

### DNA extraction and bacterial 16S rRNA Illumina sequencing

Genomic DNA was extracted directly from each sample, as well as from two negative (no-DNA template) controls, using the PowerSoil^®^ DNA Isolation Kit (MO BIO Laboratories, Carlsbad, CA, USA), according to manufacturers’ instructions, within 1 month from collection. High-throughput sequencing of the V3-V4 hypervariable region of the bacterial 16S rRNA gene was performed on an Illumina MiSeq platform according to the standard protocols with minor adjustments. Briefly, the V3-V4 region was PCR-amplified using universal primers [23], that contained the Illumina adapter overhang nucleotide sequences, using the NEBNext hot start high-fidelity DNA polymerase (New England Biolabs) and the following thermocycling protocol, using DNA 2ng/μl: 2 min at 98°C, 20 cycles of 15 s at 98°C– 30 s at 63°C– 30 s at 72°C, and a final elongation of 5 min at 72°C. Amplicons were purified using AMPure XP beads (Beckman Coulter) and the NEBNext hot start high-fidelity DNA polymerase was used for the index PCR with Nextera XT index primers (Illumina) according to the following thermocycling protocol: 3 min at 95°C, 8 cycles of 30 s at 95°C– 30 s at 55°C– 30 s at 72°C, and 5 min at 72°C. The indexed samples were purified using AMPure XP beads, quantified using the Qubit dsDNA high sensitivity kit (Life Technologies), and equal quantities from each sample were pooled. The resulting pooled library was quantified using the NEBNext library quantification kit (New England Biolabs) and sequenced using the v3 chemistry (301 bp paired-end reads). Raw sequence data have been deposited in the European Nucleotide Archive database of EMBL-EBI under accession number PRJEB21999.

### Bioinformatics and statistical analyses

Raw paired-end Illumina reads were trimmed for 16S rRNA gene primer sequences using Cutadapt (https://cutadapt.readthedocs.org/en/stable/). Pre-processed sequence data were processed using the Quantitative Insights Into Microbial Ecology (QIIME) software suite [24]. Successfully joined sequences were quality filtered in QIIME using default settings. Then, sequences were clustered into OTUs on the basis of similarity to known bacterial sequences available in the Greengenes database (v13.8; http://greengenes.secondgenome.com/; 97% sequence similarity cut-off) using the UCLUST software; sequences that could not be matched to references in the Greengenes database were clustered *de novo* based on pair-wise sequence identity (97% sequence similarity cut-off). The first selected cluster seed was considered as the representative sequence of each OTU. Then, representative sequences were assigned to taxonomy using the UCLUST software. Singleton OTUs and ‘contaminant’ sequences (from no-DNA control samples) were removed prior to downstream analyses. Total sum normalisation (TSS) was applied followed by cumulative-sum scaling (CSS) to correct biases introduced by TSS, and log2 transformation to account for the non-normal distribution of taxonomic counts data. Statistical analyses were executed using the Calypso software [25] (cgenome.net/calypso/); samples were clustered using supervised Canonical Correspondence Analysis (CCA) (including ‘infection status’ as explanatory variable). Differences in bacterial alpha diversity (Shannon diversity) and richness between groups, as well as in the abundance of individual taxa, were evaluated using paired t-test. Beta diversity was calculated using weighted UniFrac distances and differences in beta diversity were calculated using PERMDISP (Permutational Analysis of Multivariate Dispersions) through the betadisper function [26]. Differences in the composition of the faecal microbiota between groups were assessed using the LEfSe (*L*inear discriminant analysis *Ef*fect *S*iz*e*) workflow [27], by assigning infection/treatment ‘groupings’ as comparison class. Metagenome functional contents were analysed using the software package PICRUSt (v1.0.0) to predict gene contents and metagenomic functional information [28]. Sequences were aligned to data available in the Greengenes database v.13.5 and OTUs were assigned using a 97% identity cut-off. The resulting OTU table was then imported into PICRUSt and used to derive relative Kyoto Encyclopedia of Genes and Genomes (KEGG) pathway abundance [28]. Differences in KEGG pathway abundance between groups were assessed using ANOVA embedded in the software suite STAMP [29]. To minimise the risk of other variables confounding the results, binomial logistic multiple-regression models were applied to the dataset. The infection status of each study participant was used as a dependent variable and other factors including age, gender, village, education and occupation, as independent variables, including interaction terms, to identify any risk factors associated with helminth infection (cf. S1 Table).

## Results

Of 76 human volunteers enrolled in this study, 11 were positive for hookworms and/or roundworms and/or whipworms (*H*+) (Table 1), while 27 were negative despite no prior anthelmintic treatment (*H*-) (Table 1 and S1 Table). A total of 38 subjects had received regular anthelmintic treatment (*H*t) with pyrantel pamoate (Table 1 and S1 Table). Therefore, 11 samples from each of *H*- and *Ht* cohorts were selected, based on available metadata, for high-throughput sequencing of bacterial 16S rRNA and subsequent comparative analyses with samples from the *H*+ group (Table 1). Logistic multiple-regression models were applied to these samples, but none of the assessed independent variables had a significant association with infection status of the study participants. From these 33 samples, a total of 17,576,532 paired-end reads were generated (not shown) and subjected to further processing. A total of 3,694,717 high-quality sequences (per sample mean 111,960 ± 40,845) were retained after quality control. Rarefaction curves generated following *in silico* subtraction of low-quality and contaminant sequences indicated that the majority of faecal bacterial communities were represented in the remaining sequence data, thus allowing us to undertake further analyses. These sequences were assigned to 11,371 OTUs and 12 bacterial phyla.

The phyla Firmicutes (mean of 50.9%) and Bacteroidetes (mean of 39.2%) were predominant in all samples analysed, followed by the phyla Proteobacteria (mean of 3.6%) and Actinobacteria (mean of 3.0%) (S2 Fig). At the family level, Prevotellaceae (mean of 26.4%), Ruminococcaceae (mean of 24.7%), Lachnospiraceae (mean of 13.0%) and Bacteroidaceae (mean of 8.1%) were most abundant (S2 Fig). Bacteroidaceae were highly abundant in some of the *Ht* (n = 3) and *H+* (n = 2) subjects, but only in one *H-* study participant; the same samples also showed a significant reduction in Prevotellaceae (S2 Fig). The species *Prevotella copri* was abundant in the microbiota of >75% volunteers in this study and made up 7–69% (mean 17.3%) of all microbe species in these samples (S3 Fig).

**Fig 2:**
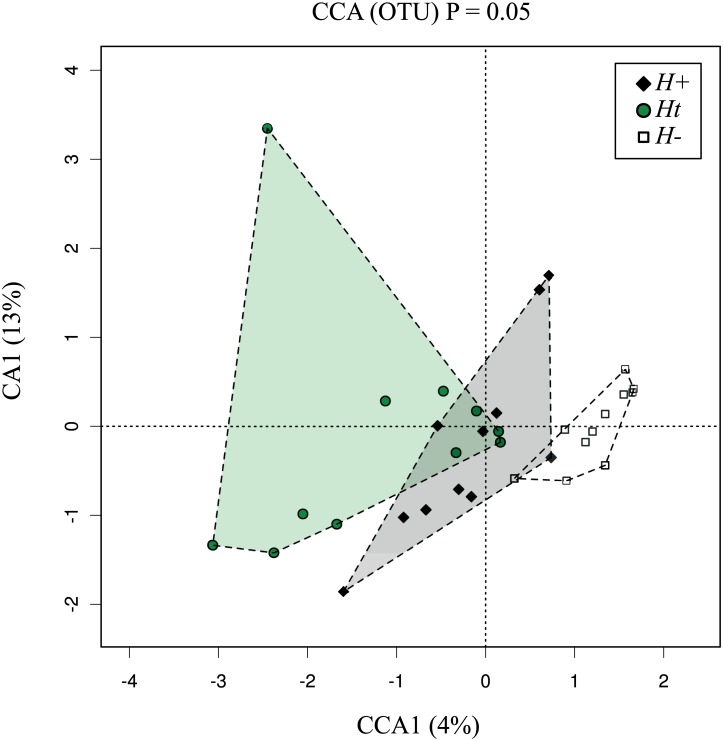
Supervised Canonical Correspondence Analysis (CCA) displaying the compositional distribution of the faecal microbiota between helminth-positive (*H*+), helminth-negative (*H*-) and helminth-negative but regularly treated (*Ht*) subjects.

Subject faecal microbial communities were ordinated by CCA, which separated samples by infection or treatment status (Fig 2) (*P* = 0.05). No significant differences in OTU alpha diversity (Shannon) and richness were recorded between groups (*P* = 0.65, *P* = 0.13) (S4 Fig).

**Fig 3:**
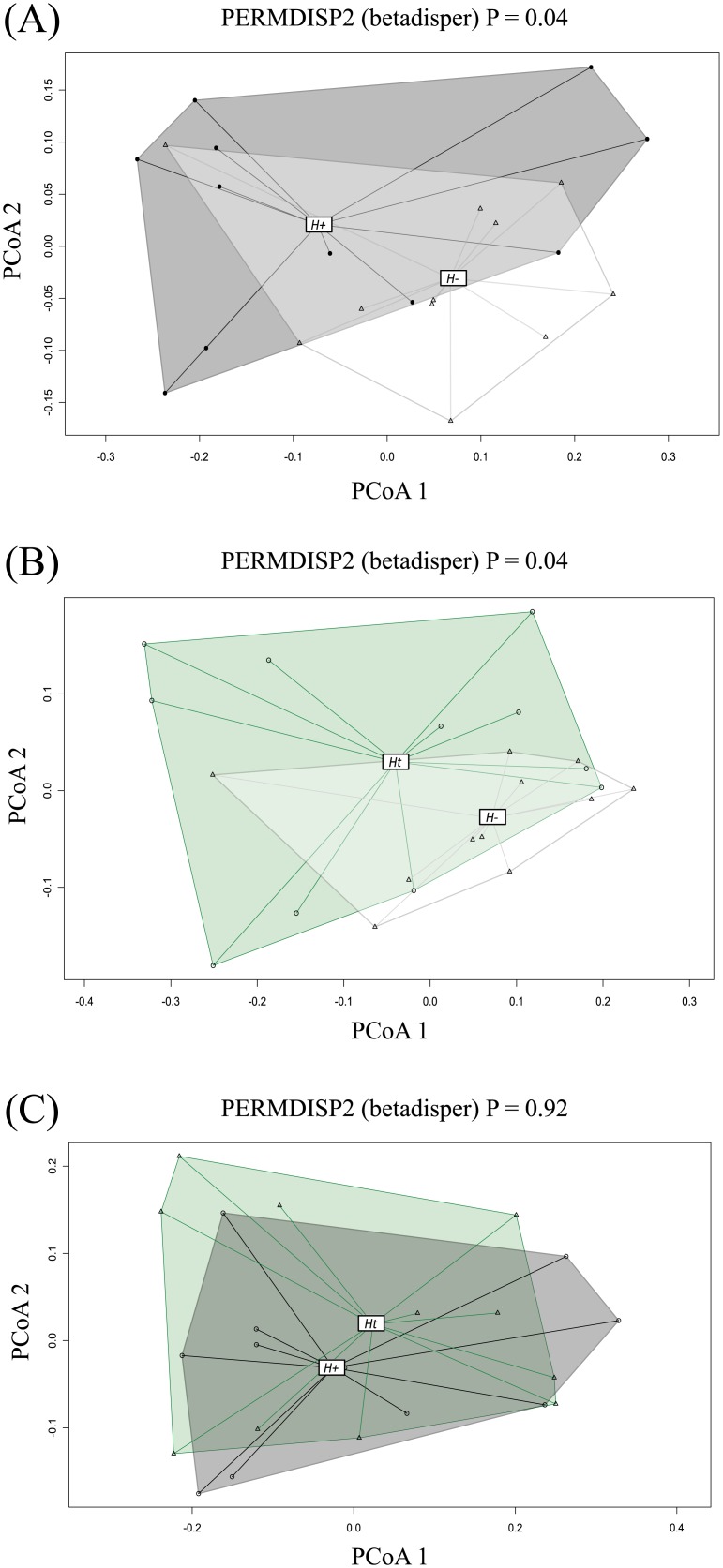
Permutational Analysis of Multivariate Dispersions indicating differences in global microbial community composition of study subjects. (A) helminth-positive (*H+*) and helminth-negative (*H-*), (B) regularly treated (*Ht*) and *H-*, and (C) *H+* and *Ht* subjects.

However, the *H+* and *Ht* microbiota was significantly more variable (i.e. characterised by increased beta diversity) compared with *H-* subjects (*P* = 0.04) (Fig 3), which indicated differences in overall heterogeneity of species composition between sample groups, rather than in overall species composition.

**Fig 4:**
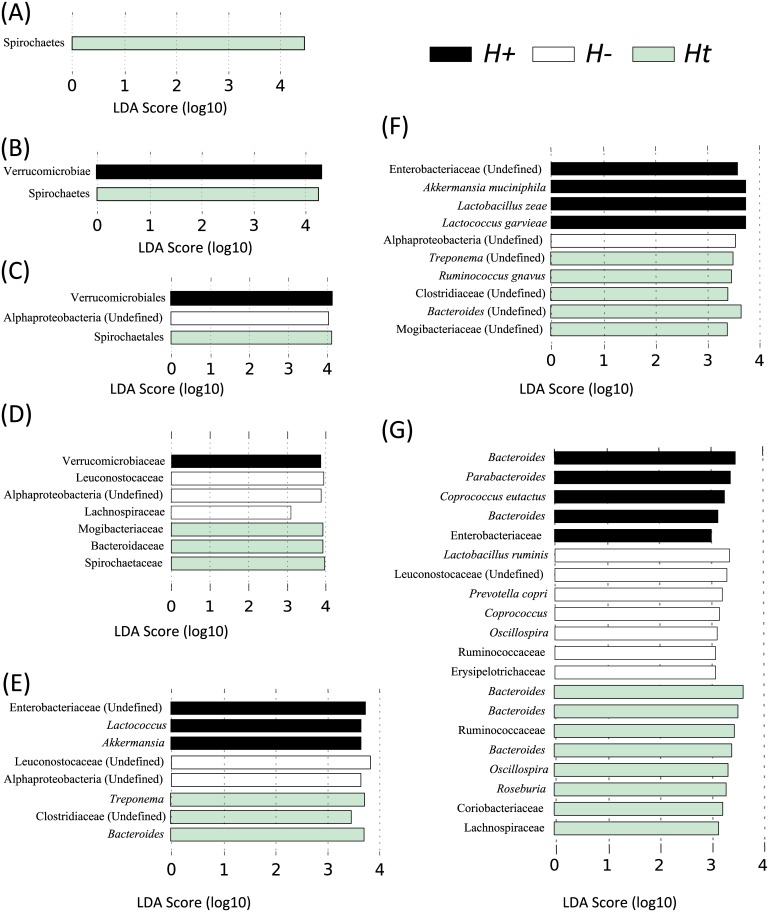
Differentially abundant faecal bacteria in helminth-positive (*H*+), helminth-negative (*H*-) and helminth-negative but regularly treated (*Ht*) subjects, based on *L*DA *Ef*fect *S*iz*e* (LEfSe) analysis. Phylum (A), Class (B), Order (C), Family (D), Genus (E), Species (F) and OTUs (G). Taxa highlighted in black/white/green indicate an overrepresentation in *H*+ /*H*- /*Ht*, respectively.

Analysis by LEfSe, also supported by ANOVA, identified differences in abundance of individual taxa at the phylum, class, order, family, genus and species level between the three groups (Fig 4). In particular, Verrucomicrobiae (Class), Verrucomicrobiales (Order), Verrucomicrobiaceae and Enterobacteriaceae (Family), *Lactococcus*, *Akkermansia* and a genus belonging to the Enterobacteriaceae (Genus) and *Akkermansia muciniphila* (Species) showed a trend towards increased abundance in *H+* compared to the other two groups (Fig 4). Compared to *H+*, Leuconostocaceae and Bacteroidaceae (Family) and *Bacteroides* (Genus), were increased in *H-* and *Ht*, respectively (Fig 4). Compared to *H+*, *Leuconostocaceae* and *Bacteroidaceae* (Family) and *Bacteroides* (Genus) were increased in *H-* and *Ht*, respectively (Fig 4).

**Fig 5:**
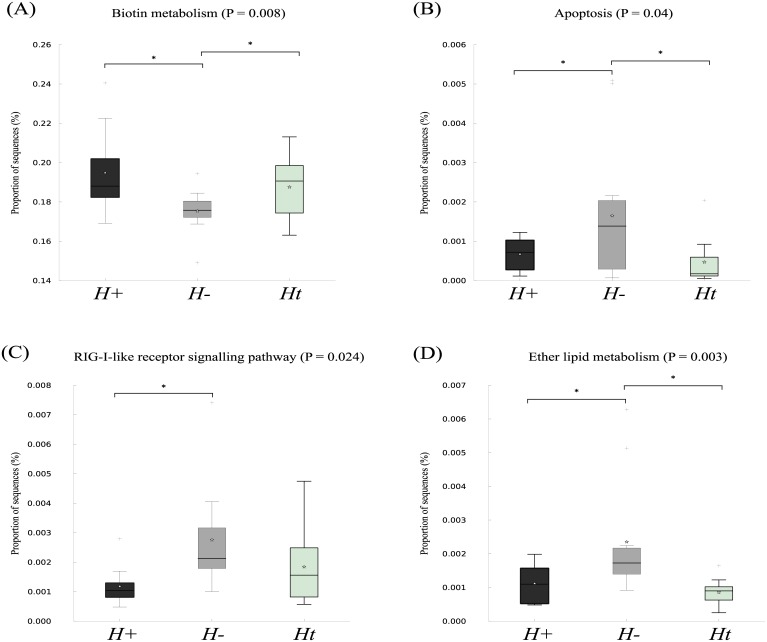
Differences in relative abundance of KEGG pathways encoded in the faecal microbiota of helminth-positive (*H*+), helminth-negative (*H*-) and helminth-negative but regularly treated (*Ht*) subjects. Biotin metabolism (A), apoptosis (B), RIG-I-like receptor signalling (C) and ether lipid metabolism (D); significant differences (< 0.05) are indicated with asterisks (*).

The same microbial metabolic and functional KEGG pathways were inferred by PICRUSt analysis in all three groups (S5 Fig). However, ‘lipid metabolism’ (*P* = 0.003), ‘rig-like receptor signalling pathway’ (*P* = 0.024), and ‘apoptosis’ (*P* = 0.04), were down-regulated in *H+* compared with *H-*/*Ht* subjects, while the ‘biotin pathway’ was upregulated in *H+* compared with *H-*/*Ht* (*P* = 0.008) (Fig 5).

## Discussion

In this study, we analysed the effects of patent infections by parasitic helminths, as well as of repeated prophylactic administrations of anthelmintics, on the composition of the faecal microbiota of human volunteers from endemic areas of Sri Lanka. Bacterial sequence data generated showed that the gut microbiota of subjects enrolled in our study was predominantly composed by species of the phylum Firmicutes, followed by those of the phyla Bacteroidetes, Proteobacteria and Actinobacteria, irrespective of infection and/or treatment status. This observation is in agreement with data from previous studies of the effects of natural or experimental helminth infections on the composition of the human gut microbiota [8, 9, 15, 17]. In particular, at the genus level, a significant proportion of most samples analysed were represented by *Prevotella* spp., which reflects the findings from a previous study conducted in a cohort of Ecuadorean children infected by roundworms [8]. *Prevotella* spp. are known to play a key role in carbohydrate metabolism [30] and thus their expansion is likely due to a high carbohydrate/fibre diet, which is common in Sri Lanka [31]. An abundance of bacteria of the genus *Prevotella* has been linked to a concomitant decrease of *Bacteroides* spp., and vice versa, likely as a consequence of the metabolic differences between these two genera [30]. Indeed, in this study, *Bacteroides* spp. were abundant in the gut microbiota of subjects from the fishing villages Valalai and Kandakuliya, whose diet is typically high in animal-derived proteins and fats [30]. Nevertheless, *Bacteroides* were also abundant in samples from volunteers from Hanthana (tea estate). However, since our questionnaire did not include detailed questions on individual dietary habits, it was not possible to speculate whether diet-related factors may have caused the observed differences in the composition of the gut microbiota of Hanthana villagers.

In this study, CCA analysis clustered samples according to infection or treatment status. However, analyses of OTU richness (i.e. the number of species within a sample) and alpha diversity (i.e. a measure of sample richness and evenness, the latter being defined as the relative abundance of individual species within a sample) detected no significant differences between *H+*, *H-* and *Ht* individuals. While this observation supports the findings from a previous study carried out in a cohort of human volunteers experimentally infected with *N*. *americanus* [18], an investigation of the impact of naturally acquired helminth infections on the composition of the gut microbiota of a cohort of Ecuadorean children [8] resulted in contrasting results. Indeed, while sole infections by *T*. *trichiura* could not be associated with detectable changes in microbial richness and alpha diversity, the gut microbiota of subjects with concomitant infections by *T*. *trichiura* and *A*. *lumbricoides* displayed a notable decrease of the latter [8]. Nonetheless, a recent study conducted in a cohort of naturally helminth-infected indigenous Malaysians [9], as well as other studies in experimentally infected individuals [15, 17], indicated an increase in alpha diversity associated with parasite infections. It is plausible to hypothesise that these varying observations could be linked, for example, to differences in sample sizes, in the ‘baseline’ composition of the gut microbiota of subjects enrolled, in parasite species causing the infections (hookworms *vs*. whipworms *vs*. ascarids), and/or in the infection stage at which samples were collected (acute and chronic in case of experimental infections *vs*. ‘undefined’ in case of natural infections). Nevertheless, since a range of GI inflammatory diseases have been associated with a decrease in microbial diversity [32–34], it has been hypothesised that a helminth-mediated increase in microbial alpha diversity may represent a potential mechanism by which helminths are able to suppress inflammation [9, 15, 17, 35]. This aspect deserves further investigation using pre-defined and standardised experimental set-ups.

Whilst we detected no differences in overall bacterial richness and alpha diversity between *H+*, *H-* and *Ht* groups, beta diversity was significantly increased in *H+* and *Ht* subjects when each of these groups were individually compared to the uninfected *H-* cohort. Unlike alpha diversity, beta diversity provides a measure of the distance or dissimilarity between pairs or groups of samples [26]. An increased beta diversity has been previously observed in the gut microbiota of veterinary species, such as mice infected with *Trichuris muris* [36], rats infected with *Hymenolepis diminuta* [37], and goats infected with *Haemonchus contortus* [38]. In humans, the higher beta diversity observed in *H+* individuals compared with the uninfected controls corroborates the hypothesis that helminth infections are accompanied by qualitative and quantitative changes in the composition of the host gut microbiota that are, based on data available thus far, inconsistent between individuals [15, 17, 18]. However, our data also suggests that anthelmintic treatment alone may be responsible for significant changes in the gut microbiota of human hosts. To date and to the best of our knowledge, no studies have shown a direct effect of anthelmintics on the composition the vertebrate gut microbiota. Nevertheless, pyrantel pamoate, the anthelmintic administered to subjects enrolled in our study, has been shown to affect, besides helminths, protozoans such as *Giardia* [39]. The microbiota profiling technique used in this study did not allow to investigate the effects that helminth infections or anthelmintic treatment exert on populations of commensal or pathogenic eukaryotes, and thus the hypothesis that repeated doses of pyrantel may have resulted in substantial modifications of such populations requires further testing.

Significant alterations of individual bacterial taxa were detected between *H+*, *H-* and *Ht* subjects. Amongst these, *Akkermansia muciniphila* (class Verrucomicrobiae) was significantly increased in *H+* individuals when compared to uninfected subjects. *A*. *muciniphila* is an anaerobic bacterium commonly detected in the human gut microbiota, where it primarily degrades host mucins [40]. Both vertebrate and helminth mucins have been shown to play key roles in the complex network of interactions occurring at the helminth-host interface [41]. For instance, the surface coat of the infective larval stage of the canine roundworm *Toxocara canis* has been shown to express high levels of a mucin-like glycoprotein (TES-120) which is shed following its binding by host antibodies or immune cells, thus suggesting that these molecules play a major role in protecting the parasite from the host immune response [42]. On the other hand, a dramatically increased production of host mucins was observed in macaques experimentally infected with the whipworm *T*. *trichiura* [35], likely as a consequence of the onset of Th2-type immunity stimulated by the infection [43]. Therefore, it may be possible that our observation of increased populations of *A*. *muciniphila* may be a direct consequence of the surge in helminth- and host-derived mucins in infected subjects. Interestingly, previous studies have shown that *A*. *muciniphila* populations are reduced in individuals with severe appendicitis and inflammatory bowel disease (IBD), which led the authors to hypothesise an anti-inflammatory role for this bacterial species [44–46]. Given the known anti-inflammatory properties of a range of helminth species [15, 17, 18, 35, 47, 48], the role of helminth-induced expansions in populations of mucin-degrading bacteria should be tested in future studies aimed at dissecting the causality of parasite-mediated suppression of inflammation.

Amongst the Firmicutes, the family *Leuconostocaceae* (order Lactobacillales) was significantly increased in *H-* subjects. These bacteria belong to the lactic acid bacteria, a major group of autochthonous microbes that reside in the gut of humans and animals and that exert immune-modulatory functions [49]. Lactic acid bacteria are known probiotics [49]; yet, recent investigations, by us and others, in humans experimentally or naturally infected by GI nematodes did not report significant associations between helminth infections and expanded populations of lactic acid bacteria [8, 9, 15, 17, 18]. Conversely, previous studies of murine models of nematode infections have shown a marked increase in populations of lactobacilli (belonging to the Lactobacillaceae, a family of lactic acid bacteria distinct from the *Leuconostocaceae* but with similar metabolic properties) in response to parasite establishment [13, 48, 50]. The specific groups of lactic acid bacteria shown to be associated with helminth infection are inconsistent between this and previous studies, which could be linked to host-specific responses to parasites; nevertheless, in the future, studies of parasite-microbiota interactions conducted on larger human and/or animal cohorts should particularly focus on this group of probiotics [49, 51].

In order to correlate data on the composition of the human gut microbiota in response to helminth infection and anthelmintic treatment to inferred changes in bacterial metabolism, we conducted a predictive metagenomics analysis using PICRUSt. Whilst inferred KEGG pathways were consistent across groups, as also shown in a similar investigation of the faecal microbiota of a helminth-infected community from Malaysia [9], ‘biotin metabolism’ was inferred to be upregulated in *H+* individuals compared to *H-*. Biotin is a B-vitamin with key roles in gene expression, cell signalling and chromatin structure [52]; in particular, biotin dependent signalling pathways regulate the expression of genes with key biological functions, e.g. apoptosis and cell survival [52]. Indeed, a significant downregulation of the KEGG pathway ‘apoptosis’ was observed in *H+* compared to *H-* volunteers. Overall, these findings emphasise that qualitative and quantitative compositional changes in helminth-infected individuals may be accompanied by significant alterations of the microbial metabolism which, in turn, may greatly impact host nutrition and immunity. Clearly, this data requires experimental validation using comprehensive metabolomics studies of the gut microbiota of helminth-infected hosts during acute and chronic infections.

## Conclusion

Data from this study augments current knowledge of the effect that helminth infections and continued prophylactic treatment exert on the composition of the gut microbiota of the human host. However, inherent limitations may have impaired our ability to detect minor changes in populations of bacteria affected by parasites and/or anthelmintic treatment. Amongst these limitations, our relatively small sample size, dictated by the prevalence of helminth infections in the Sri Lankan community enrolled in this investigation, may have affected our statistical power; in addition, dietary variabilities, as well as differences in species of infecting helminths and parasite loads, while effectively representing a ‘real world’ scenario, may have introduced a range of confounding factors that, under the circumstances of this study, we were unable to fully evaluate. Nevertheless, we detected a significantly increased beta diversity in the microbiota of *H+* compared with the *H-* counterpart, together with compositional changes in the gut microbiota of *H+*, *H-* and and *Ht* subjects, thus indicating a distinct effect of both helminth infection and continued prophylactic treatment on the host gut microbiota. In addition, we also identified potential microbial metabolic changes associated with helminth infections, which further emphasises the need for further investigations of the role/s that helminth-induced changes in bacterial metabolism play in the complex network of host-parasite interactions. Overall, our findings add valuable knowledge to the vast, and yet little explored, research field of parasite—microbiota interactions and will provide a basis for the elucidation of the role such interactions play in pathogenic and immune-modulatory properties of parasitic nematodes in both human and animal hosts.

## Supporting information

S1 Fig Questionnaire used for the collection of metadata from 76 human volunteers screened for the presence of patent infections by gastrointestinal nematodes. (EPS)

S2 Fig Composition of the faecal microbiota of helminth-positive (*H+*), helminth-negative (*H-*) and helminth-negative but regularly treated (*Ht*) subjects at the Phylum (A) and Family (B) level. (EPS)

S3 Fig *Prevotella copri* abundance in helminth-positive (*H+*), helminth-negative (*H-*) and helminth-negative but regularly treated (*Ht*) subjects. (EPS)

S4 Fig Differences in overall taxonomic species richness (A) and diversity (B) between the faecal microbiota of helminth-positive (*H+*), helminth-negative (*H-*) and helminth-negative but regularly treated (*Ht*) subjects. (EPS)

S5 Fig Relative abundances of KEGG pathways encoded in the gut microbiota of helminth-positive (*H+*), helminth-negative (*H-*) and helminth-negative but regularly treated (*Ht*) subjects, determined by inferred metagenomic analyses with PICRUSt. (EPS)

S1 Table Metadata associated with helminth-positive (*H+*), helminth-negative (*H-*) and helminth-negative but regularly treated (*Ht*) subjects enrolled in the study. (XLSX)
